# Clinical Pathways and Outcomes of Andexanet Alfa Administration for the Reversal of Critical Bleeding in Patients on Oral Direct Factor Xa Inhibitors

**DOI:** 10.1055/a-2306-0804

**Published:** 2024-05-13

**Authors:** Mark Goldin, Kolton Smith, Ioannis Koulas, Tungming Leung, Mayuri Ravi, Sanjit Parhar, Sejal Shah, Kayla Floyd, Lori Ohanesian, Rachel Bain, Daniella Defonte, Kanta Ochani, Amanda Lin, Bhumi Patel, Nikolaos Tsaftaridis, Jack Jnani, Alex C. Spyropoulos

**Affiliations:** 1 Institute of Health System Science, Feinstein Institutes for Medical Research, Northwell Health, Manhasset, New York, United States; 2 Department of Medicine, Donald and Barbara Zucker School of Medicine at Hofstra/Northwell, Hempstead, New York, United States; 3 Department of Medicine, Lenox Hill Hospital at Northwell Health, New York, New York, United States; 4 Biostatistics Unit, Office of Academic Affairs, Northwell Health, Hempstead, New York, United States; 5 Department of Medicine, North Shore University Hospital, Manhasset, New York, United States; 6 Clinical Pharmacy, Long Island Jewish Medical Center, New Hyde Park, New York, United States; 7 Clinical Pharmacy, Long Island Jewish Valley Stream, Valley Stream, New York, United States; 8 Clinical Pharmacy, Glen Cove Hospital, Glen Cove, New York, United States; 9 Clinical Pharmacy, North Shore University Hospital, Manhasset, New York, United States

**Keywords:** DOAC, critical bleeding, andexanet alfa, clinical pathway, thromboembolism

## Abstract

**Background** Andexanet is U.S. Food and Drug Administration (FDA) approved for the reversal of critical bleeding from factor Xa inhibitors and off-label for surgical reversal. Data are lacking on andexanet administration processes.

**Methods** We retrospectively studied patients at a 23-hospital system who received andexanet from November 2019 to March 2023. Abstractors coded demographics, comorbidities, anticoagulant use, andexanet indication, and process times. The primary outcome was presentation-to-andexanet time; diagnosis, ordering, and administration times were calculated. Secondary outcomes included in-hospital postandexanet major thromboembolism/bleeding and mortality.

**Results** In total, 141 patients were analyzed. Andexanet indications were predominantly neurologic bleeding (85.8%). Twenty-four patients (17.0%) were transferred from nontertiary/academic centers to tertiary/academic centers. The median presentation-to-administration time was 192.5 minutes (interquartile range [IQR]: 108.0–337.0 minutes). Components were as follows: 72.5 minutes (IQR: 39.0–137.5 minutes) for bleeding diagnosis; 35.5 minutes (IQR: 0–96.5 minutes) for andexanet ordering; and 53.0 minutes (IQR: 38.5–78.5 minutes) for administration, which was longer at tertiary/academic hospitals (ratio 1.5, 95% confidence interval [CI]: 1.2–2.0, *p* = 0.002). Gastrointestinal or other critical bleeding (ratio 2.59, 95% CI: 1.67–4.02, *p* < 0.001), and tertiary/academic center treatment (ratio 1.58, 95% CI: 1.15–2.18, *p* = 0.005), were associated with increased time. Major thromboembolism, bleeding, and mortality occurred in 10.6, 12.0, and 22.9% of patients, respectively.

**Conclusions** In our cohort, the median presentation-to-administration time was over 3 hours. Cumulative times were longer at tertiary/academic hospitals and for gastrointestinal/other bleeding. Postandexanet major thromboembolism/bleeding occurred more at tertiary/academic hospitals, possibly related to transfers. Prospective studies may elucidate clinical decision-making bottlenecks.

## Introduction

Andexanet alfa is a recombinant protein similar in structure to endogenous factor Xa (FXa) that acts as a decoy in binding FXa receptors to counteract direct oral anticoagulants (DOACs). It was the first agent approved by the U.S. Food and Drug Administration (FDA) for the reversal of critical bleeding associated with apixaban and rivaroxaban. 1 Efficacy and safety of andexanet alfa was demonstrated in the ANNEXA-4 trial, 2 which enrolled 479 patients with major bleeding (including 69.1% intracranial hemorrhage [ICH] and 22.8% gastrointestinal bleeding) who had taken a FXa inhibitor within the preceding 18 hours. The final study population showed excellent or good hemostasis in 80% of evaluated patients, compared with 72% in a previous study of prothrombin complex concentrate (PCC) in patients with bleeding while taking vitamin K antagonist therapy. 3 Moreover, rates of postandexanet thrombosis and death were comparable to those in previous studies of patients who stopped FXa inhibitors due to acute bleeding. 4 5 These findings, along with data showing less mortality benefit when using PCCs, 2 5 led professional society expert panels to recommend andexanet alfa for the reversal of critical bleeding in patients on oral direct FXa inhibitors. 6 7 8

A recently published study of time to anticoagulation reversal and outcomes in ICH suggested a 1-hour door-to-treatment time or less as being optimal for improved survival. 9 In addition, a “call-to-action” for mandating a multidisciplinary and process-centered “Code ICH” also suggested a 1 hour window for the reversal of anticoagulation in the setting of ICH. 10 As such, it has been suggested that prehospital protocols and early administration of andexanet in cases of life-threatening bleeding should be prioritized to reduce morbidity and mortality. 11 To date, data are lacking on the optimal processes at a health care system level to assess administration times of andexanet alfa. Our study aimed to evaluate institutional processes, hospital types, and clinical outcomes regarding reversal with andexanet alfa for patients on oral direct FXa inhibitors with critical bleeding.

## Methods

### Study Oversight

This was an investigator-initiated, retrospective, observational study conducted at a large, 23-hospital, integrated health care delivery network in the New York metropolitan area, supported by AstraZeneca Pharmaceuticals, PC. The funders had no role in study design or conduct, data collection or analysis, or writing of the manuscript. The study was approved by the health system Institutional Review Board overseeing all study hospitals with a waiver of informed consent for this retrospective, noninterventional study. Authors assume responsibility for the accuracy and completeness of the data and analyses.

### Patients and Data Collection

Clinical pharmacy colleagues compiled and maintained a database using REDCap (Research Electronic Data Capture) 12 13 of all patients admitted to health system hospitals who received andexanet alfa for the reversal of oral direct FXa inhibitor critical bleeding starting from November 3, 2019, when andexanet alfa was first added to our formulary, through March 4, 2023. Our system developed a centralized protocol that came into place at the time of formulary approval for the use of andexanet in critical site bleeding related to ICH, whereas use for other critical site bleeding and limited neurosurgical procedures required a medical director or designee approval. The protocol is included in the Supplementary Appendix (available in the online version). A dedicated team of abstractors manually collected demographics and other characteristics including age, sex, race, weight, estimated glomerular filtration rate (eGFR), baseline cardiovascular conditions (including history of myocardial infarction, ischemic stroke, deep vein thrombosis [DVT], pulmonary embolism [PE], atrial fibrillation, congestive heart failure, diabetes mellitus, and hypertension), type of DOAC (i.e., apixaban or rivaroxaban), indication for anticoagulation, total daily anticoagulant dose, and time of last dose prior to presentation to the emergency department (ED). We also collected data on concomitant use of antiplatelet agents (e.g., aspirin and P2Y12 inhibitors). We recorded bleeding indications for reversal with andexanet alfa, which included ICH, other central nervous system (CNS) bleeding, gastrointestinal bleeding, and other critical site bleeding (which included intraocular hemorrhage with vision compromise, airway bleeding/pulmonary hemorrhage, hemopericardium, aortic rupture/dissection/hemorrhage, closed space hemorrhage/compartment syndrome risk, hepatic artery bleeding, and bleeding due to perforated viscus). We also coded cases in which the indication for andexanet was an urgent surgical reversal. Finally, each patient was categorized as receiving treatment at a tertiary/academic center versus a nontertiary/academic center; the number of patients transferred from nontertiary/academic centers to tertiary/academic centers was tallied.

### Outcomes

The primary outcome was cumulative process time from patient presentation to the ED to administration of andexanet alfa. This cumulative time was divided into three segments. The first segment was time from patient presentation to the ED until diagnosis of critical bleeding. Diagnosis time was generally coded as the time a final radiologic report was submitted in the electronic health record (EHR), in cases that depended on imaging for confirmation of bleeding, for example, ICH, other central nervous system bleeding, and hemopericardium. In cases of gastrointestinal bleeding, diagnosis time was coded as the time of EHR documentation by a specialist recommending a confirmatory study (e.g., endoscopy). The second segment was time from diagnosis of critical bleeding to ordering of andexanet alfa within the EHR, and the final segment was time from ordering of andexanet alfa to administration of andexanet alfa (as documented by nursing staff within the medication administration record of the EHR). Manual chart review revealed frequent instances in which patients were presumptively diagnosed with critical bleeding, and andexanet alfa was ordered before radiologic studies were obtained. In these cases, time from diagnosis of critical bleeding to andexanet alfa ordering was coded as zero.

Secondary outcomes were clinical and included a composite of in-hospital postandexanet venous thromboembolism (VTE) (including DVT, PE, or splanchnic vein thrombosis); arterial thromboembolism (ATE) (including myocardial infarction, acute ischemic stroke, systemic embolism, or major adverse limb event); and major bleeding, defined according to the International Society on Thrombosis and Haemostasis criteria, 14 and in-hospital mortality.

### Statistical Analysis

Descriptive statistics (median, interquartile range [IQR], minimum, maximum for continuous variables, and frequency distribution for categorical variables) were calculated. Univariable linear regression was used to screen variables which were associated with the log-transformed cumulative time from patient presentation to andexanet administration with a *p* -value criterion of *p* < 0.05 for entry into the model selection procedure. Backward selection was used with variable entry and retention criteria of *p* < 0.05 to select the final multivariable model. This procedure was also used to screen variables associated with the three time segments of interest (diagnosis, ordering, and administration times). Log transformation was performed to meet the assumption of the regression model. We included sensitivity analyses where these time segments were analyzed separately for tertiary versus nontertiary hospitals. Univariable logistic regression was also used to screen variables which were associated with the composite secondary outcomes of postandexanet alfa ATE/VTE/major bleeding or in-hospital mortality with a *p* -value criterion of *p* < 0.05 for entry into the model selection procedure. Backward selection was used with variable entry and retention criteria of *p* < 0.05 to select the final multivariable model.

## Results

### Characteristics of the Population

The initial study population consisted of 165 patients. In 13 cases, technical limitations precluded extraction of EHR data from outside our system's main facilities. An additional six patient records were excluded for lack of a documented ED arrival time. Finally, five patient records were excluded for unreliable component times. After a manual review of the remaining 141 records, there were rare instances of missing data: baseline anticoagulant dose was not captured in approximately 10 cases, time of diagnosis in approximately four cases, and vital status was unknown in one case. Thus, the final study population consisted of 141 patients: mean age was 77.3 years; 56.7% of patients were men; self-identified race breakdown was 73.8% white, 8.5% black or African American, 2.1% Asian, and 14.2% other/multiracial; mean weight was 79.6 kg; and mean eGFR was 65.7 mL/min/1.73 m ^2^ ( Table 1 ). At baseline, 73.8% of patients were prescribed apixaban and 25.5% rivaroxaban; 31.2% of patients were categorized as taking a low DOAC dose, and 68.1% a high (treatment) DOAC dose. Most patients (66.7%) had taken their most recent dose of anticoagulant within 24 hours preceding ED presentation. Concomitant single antiplatelet therapy had been prescribed in 34.8% of patients (27.7% aspirin and 6.4% clopidogrel); only 4.3% of patients had been prescribed dual antiplatelet therapy at baseline.

**Table 1:** Demographics and other characteristics of cohort (N = 141)

Age in years, mean (SD)	77.3 (10.5)
Sex (%)	
Male	56.7
Female	43.3
Race, %	
Asian	2.1
Black/African American	8.5
White	73.8
Other/Multiracial	14.2
Weight in kg, mean (SD)	79.6 (22.5)
Estimated GFR in mL/min/1.73 m ^2^ , mean (SD)	65.7 (26.0)
Baseline anticoagulant use, %	
Apixaban	73.8
Rivaroxaban	25.5
Anticoagulant total daily dose, %	
Low (Apixaban ≤ 5 mg OR Rivaroxaban ≤ 10 mg)	31.2
High (Apixaban > 5 mg OR Rivaroxaban > 10 mg)	68.1
Time of last anticoagulant dose before andexanet, %	
≤ 24 h	66.7
> 24 h	33.3
Baseline antiplatelet use, %	
Aspirin	27.7
Clopidogrel	6.4
Aspirin and clopidogrel	4.3
No antiplatelets	61.7
Other reversal agent administered, % a	17.9
Number of baseline cardiovascular risk factors, %	
0–1	13.5
2–3	73.1
≥ 4	13.5
Indication for reversal, % b	
Intracranial hemorrhage	83.0
Other central nervous system bleeding	2.8
Gastrointestinal bleeding	2.1
Other critical site bleeding	9.9
Presurgery	5.0
Type of facility, %	
Tertiary/academic	72.3
Nontertiary/academic	27.7
Transfers from nontertiary/academic to tertiary/academic facilities, no. (%)S	24 (17.0)

Abbreviations: eGFR, estimate glomerular filtration rate; SD, standard deviation.

a Includes KCentra, fresh frozen plasma, desmopressin, tranexamic acid.

b Sum > 100% as some patients received andexanet and underwent surgery to treat bleeding.

Indications for andexanet alfa were predominantly neurological, with 83.0% for ICH and 2.8% for other CNS bleeding. Gastrointestinal bleeding comprised 2.1% of indications. Additional reversal agents were given in 17.9% of patients and included fresh frozen plasma, desmopressin, tranexamic acid, and KCentra (a PCC, which was given to 10 patients). Most patients (86.6%) had at least two cardiovascular comorbidities at baseline and 13.5% had at least four ( Table 1 ).

Approximately three-quarters of patients (72.3%) were treated at tertiary/academic centers; 24 patients (17.0%) were transferred from nontertiary academic centers to receive definitive treatment at tertiary/academic centers ( Table 1 ).

### Primary Outcome

For the full study population, the median cumulative time from patient presentation in the ED to andexanet alfa administration was 192.5 minutes (IQR 108.0–337.0 minutes). The median time from ED presentation to diagnosis of critical bleeding was 72.5 minutes (IQR: 39.0–137.5 minutes). The median time from diagnosis of critical site bleeding to andexanet alfa ordering was 35.5 minutes (IQR: 0–96.5 minutes). The median time from andexanet alfa ordering to andexanet alfa administration was 53.0 minutes (IQR: 38.5–78.5 minutes) ( Table 2 ).

**Table 2:** Major process and clinical outcomes

Primary outcome	
Median cumulative time from ED presentation to andexanet administration, min (IQR)	192.5 (108.0–337.0)
Median time from ED presentation to diagnosis, min (IQR)	72.5 (39.0–137.5)
Median time from diagnosis to andexanet alfa order, min (IQR)	35.5 (0–96.5)
Median time from andexanet alfa order to administration, min (IQR)	53.0 (38.5–78.5)
Secondary outcomes	
Composite of post-andexanet VTE a , ATE b , or major bleeding c , %	22.7
VTE or ATE	10.6
Major bleeding	12.0
In-hospital mortality (full population), %	22.9
Intracranial hemorrhage	22.4
Other central nervous system bleeding	25.0
Gastrointestinal bleeding	33.3
Other critical site bleeding	25.0
Presurgery	0

Abbreviations: ATE, arterial thromboembolism; ED, emergency department; IQR, interquartile range; min, minutes; VTE, venous thromboembolism.

a VTE includes deep vein thrombosis and pulmonary embolism.

b ATE includes myocardial infarction, ischemic stroke, systemic embolism, and major adverse limb event.

c Major bleeding per the International Society on Thrombosis and Haemostasis definition.

Median cumulative time from presentation to andexanet administration was significantly longer at tertiary/academic hospitals (223.0 minutes, IQR: 142.0–358.0 minutes) than at nontertiary/academic hospitals (130.0 minutes, IQR: 87.0–253.0 minutes) (ratio 1.6, 95% CI: 1.2–2.3, *p* = 0.005). There was no difference in the median time from ED presentation to the diagnosis of critical bleeding (90.0 minutes [IQR: 39.0–162.0 minutes] vs. 65.0 minutes [IQR: 39.0–98.0]) (ratio 1.41, 95% CI: 0.91–2.17, *p* = 0.122) and no difference in the median time from the diagnosis of critical site bleeding to andexanet alfa ordering (45.0 minutes [IQR: 0–98.0 minutes] vs. 16.0 minutes [IQR: 0–82.0 minutes]) (ratio 1.40, 95% CI: 0.62–3.16, *p* = 0.418) when comparing tertiary/academic hospitals versus nontertiary/academic hospitals. Longer times from andexanet alfa ordering to administration were observed at tertiary/academic hospitals (median 59.0 minutes, IQR: 46.0–82.0 minutes) compared with nontertiary/academic hospitals (median 39.0 minutes, IQR: 32.0–62.0 minutes) (ratio 1.5, 95% CI: 1.2–2.0, *p* = 0.002) ( Supplemental Table S1 , available in the online version).

### Secondary Outcomes

Postandexanet major thromboembolism, bleeding, and mortality occurred in 10.6, 12.0, and 22.9% of the overall population, respectively ( Table 2 ). The composite of postandexanet major thromboembolism or bleeding occurred more often in patients at tertiary/academic centers (29.4 vs. 5.1%, odds ratio [OR] 7.71, 95% CI: 1.75–34.04, *p* = 0.007). This was driven by numerical but nonsignificant differences in thromboembolism (14.7 vs. 0%, *p* = 0.07). There was no difference in postandexanet alfa major bleeding or in-hospital mortality ( Supplemental Table S1 , available in the online version).

### Regression Analyses

Age, sex, race, time from last anticoagulant dose, baseline antiplatelet use, other reversal agent use, category of andexanet alfa indication (ICH or other neurologic bleeding, gastrointestinal or other critical site bleeding, or urgent surgery), and treatment at a tertiary/academic versus nontertiary academic hospital were screened for association with cumulative process time and the three time segments of interest. Our final linear regression model showed that diagnoses of gastrointestinal bleeding or other critical site bleeding (ratio 2.59, 95% CI 1.67–4.02, *p* < 0.001), as well as treatment at a tertiary/academic center (ratio 1.58, 95% CI: 1.15–2.18, *p* = 0.005), were risk factors for the increased cumulative time from ED presentation to andexanet alfa administration. None of the tested factors were associated with increased individual time segments.

Age, sex, race, weight, eGFR, type of baseline anticoagulant, total daily anticoagulant dose, time from last anticoagulant dose, baseline antiplatelet use, other reversal agent use, number of baseline cardiovascular risk factors, category of andexanet alfa indication (ICH or other neurologic bleeding, gastrointestinal or other critical site bleeding, or urgent surgery), and time segments of interest (time from ED presentation to critical bleeding diagnosis, time from diagnosis to andexanet alfa order, and time from andexanet alfa order to andexanet alfa administration) were screened for association with postandexanet thromboembolism or major bleeding. None of these factors showed a significant association with the composite clinical outcome.

## Discussion

This retrospective study of andexanet alfa use for the reversal of critical bleeding associated with oral direct FXa inhibitors evaluated process times as well as clinical outcomes for 141 patients at both tertiary/academic and nontertiary/academic facilities within a 23-hospital system. The median cumulative time from patient presentation to the ED until dosing of andexanet alfa was approximately 3 hours. Final linear regression showed that care at a tertiary/academic facility conferred 58% increased cumulative time, while gastrointestinal or other critical site bleeding conferred 2.5 times increased cumulative time. The median time from andexanet alfa order to administration was 20 minutes longer at tertiary/academic facilities, a significant difference. The secondary, composite clinical outcome of postandexanet thromboembolism or major bleeding occurred more often at tertiary/academic centers, but there was no difference in mortality.

The diagnosis phase comprised the component time with the longest median duration, at 72.5 minutes; this is despite the fact that in some instances, patients were presumptively diagnosed with critical bleeding based on the initial clinical data, before radiographic studies results were officially reported. We suspect this was due to radiology staff contacting the ordering providers to verbally convey critical imaging results in advance of submitting formal EHR reports, which historically has been common within our institution. Overall, this suggests that our data overestimate diagnosis times. Conversely, the time from diagnosis of critical bleeding to andexanet alfa order was the shortest time component, at 35.5 minutes. The above assumption thus would imply that our data underestimate ordering time. Per our institutional policy ( Supplementary Appendix , available in the online version), andexanet alfa is a restricted medication and formal approval from site designees (typically the hospital Medical Director) must be obtained in advance of ordering. We suspect this approval is the limiting step within the diagnosis-to-order time segment. Finally, the median order-to-administration time was nearly 1 hour. There are many steps within this time segment that are not typically documented within our EHR, including pharmacy verification of order, compounding, and delivery to the point of care. Moreover, it has been observed historically that the time of documentation of medication administration varies widely among nursing staff and, in some cases, occurs well after a drug has been administered. This implies our data overestimate this time segment. Differences in these pharmacy or nursing processes may also account for longer order-to-administration times at tertiary/academic hospitals in our study. Finally, the fact that gastrointestinal or other critical site bleeding conferred 2.5 times increased cumulative time likely relates to our institutional policy that prioritizes ICH as the preferred indication for andexanet alfa and does not include gastrointestinal bleeding, which in our system can typically be treated with other reversal agents, such as PCCs. Considering that gastrointestinal or other critical site bleeding comprised less than 15% of the cohort, process differences at tertiary/academic versus nontertiary/academic facilities are likely of greater clinical significance.

The increased frequency in clinical outcomes at tertiary/academic hospitals was driven by rates of thromboembolism, which may reflect greater severity of illness or baseline risk related to cardiovascular comorbidities in these facilities. This may also relate to frequent transfers of high-acuity patients from nontertiary community hospitals to the tertiary/academic hub of our system. However, considering the predominance of ICH, the rate of thromboembolism in the full population (10.6%) was comparable to that of an ICH substudy population of the ANNEXA-4 trial (9.3%), albeit with greater ICH-related mortality in our study (22.4 vs. 15.0%). 15

Few studies have reported process times for andexanet alfa administration. In a single-center cohort of 44 patients receiving andexanet alfa within 24 hours of surgery, the median time from andexanet alfa order to administration was 30 minutes (IQR: 19.8–43.0), while cumulative time from hospital presentation to andexanet administration was 2.6 hours (IQR: 1.2–5.5), 16 similar to the process time in our study. One additional study comparing andexanet and 4-factor PCC for ICH found longer median times from order entry to administration with andexanet (1.1 hours [IQR 0.8–1.3] vs. 0.5 hours [0.1–0.8]), which was attributed to drug compounding complexity. 17 In a descriptive, preliminary analysis of the ANNEXA-4 study that analyzed 67 patients, mean time from ED presentation to andexanet administration was 4.8 ± 1.8 hours, 18 a relatively longer process time in the context of randomized controlled trial protocol compared with our study results.

A study of health facility protocols and restrictions on andexanet alfa administration may help elucidate differences in process times. A broad effort 19 describing 89 sites within the United Kingdom National Health System found that 58% had formal andexanet protocols in place, all of which specified gastrointestinal bleeding as the only approved indication. Of these protocols, 70% contained guidance on the timing of the last DOAC dose. Authors highlighted the practice of gatekeeping, in which andexanet ordering required the approval of a specialist; this was a hematologist in 43%, a gastroenterologist in 14%, and both specialists in 4% of centers. While reasonable for judicious administration of a costly drug, gatekeeping also self-evidently increases clinical decision-making times. In our institution, typically a physician administrator served as the gatekeeper. Medical directors at study hospitals were most commonly intensivists, hospitalists, or emergency medicine specialists; to our knowledge, none were primarily neurologists, hematologists, or gastroenterologists. It is possible that gatekeeper specialty training, as well as proximity to front-line medical care, would influence process efficiency.

Broadly, the data and our experience suggest several possible explanations for differences in overall process times when comparing academic and nonacademic centers. First, as noted, the transfer rate of 17% suggests that a substantial proportion of academic center patients required additional time for evaluation and vetting of information from the transferring (nonacademic) hospital. In our experience, it is typical that additional handoffs between providers and our system's transfer center may reduce efficiency. Second, variable adherence to our system andexanet policy may have contributed; nonacademic centers may be less aware of the policy and thus less likely to wait for site approvals.

Our study has several strengths and limitations. While our data are retrospective and from a single health system, the sample population is relatively large for a study of clinical processes. Data were also acquired from nearly two dozen facilities, both tertiary/academic and nontertiary/academic, over a period of 4 years. Moreover, our study period began approximately 1 year following FDA approval of andexanet alfa, when experience with the drug was limited worldwide, and difficulty with generalizability could reasonably apply to data from any center. There were substantial challenges in calculating component times due to the organization of our EHR, as well as a historical lack of granularity in staff documentation and reliance on verbal communication of imaging results. However, as nearly five in six cases analyzed were ICH or other central nervous system bleeding, the majority of cases were coded strictly according to radiologic report times, which presumably were entered into the EHR shortly after verbal communication of critical results.

While our findings suggest that individual centers may present different bottlenecks after patients have arrived, the presence of both prehospital procedures and the use of multidisciplinary teams that already exist for critical medical emergencies such as Code Stroke may improve overall process times and clinical outcomes. A “call-to-action” using an existing hospital-based multidisciplinary team to initiate a “Code ICH” has recently been proposed using bundled care and defined metrics to initiate timely treatment of ICH, including anticoagulant-related reversal using specific and nonspecific reversal agents. 10 This type of intervention is supported by recently published data from the American Heart Association Get With The Guidelines-Stroke registry, which revealed that patients with anticoagulation-associated ICH who received reversal agents within 60 minutes of hospital arrival had decreased mortality or discharge to hospice, although no difference in functional outcome was observed. 9

In conclusion, our cohort median time from ED presentation to andexanet alfa administration was approximately 3 hours. Cumulative times were longer at tertiary/academic hospitals, which may relate to delays between drug ordering and administration. Gastrointestinal bleeding was associated with longer process time, likely due to more nuanced decision-making in light of system policy favoring andexanet for ICH. Postandexanet thromboembolism or major bleeding occurred more often at tertiary/academic hospitals, likely due to transfers of high-acuity patients. Prospective studies may elucidate pharmacy, nursing, or clinical decision-making bottlenecks. Prehospital procedures involving activation teams for ICH or other critical bleeding in the community and in-hospital multidisciplinary “Code ICH” teams may be practical strategies for improving efficiency.

## Notes

**What is known on this Topic?**

- Andexanet alfa is approved by the U.S. Food and Drug Administration (FDA) for the reversal of critical bleeding associated with apixaban and rivaroxaban.
- Prompt administration of andexanet alfa is essential for reducing morbidity and mortality.
- Few previous studies have reported times from patient presentation to andexanet alfa administration, and thus, relatively little is known about optimal processes at a health system level.

**What does this Paper Add?**

- This study showed that in a cohort of 141 patients from a 23-hospital integrated health system, median cumulative process time from patient presentation to andexanet alfa administration was more than 3 hours; time from presentation to diagnosis of critical bleeding, as well as time from andexanet alfa order to administration, represented longer component times and potential process bottlenecks.
- Care at a tertiary/academic center, or presentation with gastrointestinal bleeding were associated with longer cumulative process times, likely related to variable facility adherence to health system andexanet alfa protocols.
- Postandexanet major thromboembolism or bleeding occurred more often at tertiary/academic centers, but there was no difference in mortality.
