# Supplementary material for: Clinical Pathways and Outcomes of Andexanet Alfa Administration for the Reversal of Critical Bleeding in Patients on Oral Direct Factor Xa Inhibitors
Source: TH Open. 2024 May 13;8(2):e209–15. doi: 10.1055/a-2306-0804 (PMC11090682; doi:10.1055/a-2306-0804)
Supplement: Supplementary file 1 — Supplementary Material [file 10-1055-a-2306-0804-s24030009.pdf]

**Supplemental Table S1** Major process and clinical outcomes by the type of facility

| Primary outcome | Tertiary | Nontertiary | *p*-Value |
|---|---|---|---|
| Median cumulative time from ED presentation to andexanet administration, min (IQR) | 223.0 (142.0–358.0) | 130.0 (87.0–253.0) | 0.005 |
| Median time from ED presentation to diagnosis, min (IQR) | 90.0 (39.0–162.0) | 65.0 (39.0–98.0) | 0.122 |
| Median time from diagnosis to andexanet alfa order, min (IQR) | 45.0 (0–98.0) | 16.0 (0–82.0) | 0.418 |
| Median time from andexanet alfa order to administration, min (IQR) | 59.0 (46.0–82.0) | 39.0 (32.0–62.0) | 0.002 |
| Secondary outcomes | | | |
| Composite of postandexanet VTE[a], ATE[b], or major bleeding[c], % | 29.4 | 5.1 | 0.007 |
| VTE or ATE | 14.7 | 0 | 0.072 |
| Major bleeding | 14.7 | 5.1 | 0.173 |
| In-hospital mortality, % | 20.1 | 28.2 | 0.351 |

Abbreviations: ATE, arterial thromboembolism; ED, emergency department; IQR, interquartile range; min, minutes; VTE, venous thromboembolism.
[a]VTE includes deep vein thrombosis and pulmonary embolism.
[b]ATE includes myocardial infarction, ischemic stroke, systemic embolism, and major adverse limb event.
[c]Major bleeding per the International Society on Thrombosis and Haemostasis definition.

 © 2024. The Author(s).
