# Supplementary material for: Clinical Pathways and Outcomes of Andexanet Alfa Administration for the Reversal of Critical Bleeding in Patients on Oral Direct Factor Xa Inhibitors
Source: TH Open. 2024 May 13;8(2):e209–15. doi: 10.1055/a-2306-0804 (PMC11090682; doi:10.1055/a-2306-0804)
Supplement: Supplementary file 2 — Supplementary Appendix [file 10-1055-a-2306-0804-s24030009a.pdf]

System Pharmacy & Therapeutics

POLICY/GUIDELINE TITLE: Andexanet alfa (Andexxa®) for Reversal of Oral Factor Xa Inhibitors due to Life-threatening Bleeding or Emergency Neurosurgical Procedures	SYSTEM POLICY AND PROCEDURE MANUAL
**POLICY #:** PHT.454	**CATEGORY SECTION:** Clinical Practice Guidelines – Coalition of Leadership on Thrombosis (CLOT)
**System Approval Date:** ❖6/28/2021<br><br>**Site Implementation Date:** ❖6/28/2021	**Effective Date:** 1/6/2020<br><br>**Last Reviewed/Approved:** 1/6/2020
**Prepared by:** CLOT Council	**Notation(s):** N/A

GENERAL STATEMENT of PURPOSE

Andexanet alfa (Andexxa®) works by acting as a decoy for Factor Xa (FXa) inhibitors in the blood, thereby preventing them from inhibiting the activity of native Factor Xa. As a result, the native Factor Xa is available to participate in the coagulation process and restore hemostasis (normal clotting).

Andexanet is indicated for urgent reversal of rivaroxaban- or apixaban-associated life-threatening or uncontrolled bleeding. Additionally, retrospective case reports/case series have been published describing favorable clinical experience with andexanet use in the emergent/urgent surgical population, including neurosurgery. The intent of this policy is to provide guidance to prescribers for appropriate ordering of Andexanet, as well as dosing and monitoring recommendations.

Andexanet has not been shown to be effective for, and is not indicated for, the treatment of bleeding related to any Factor Xa inhibitors other than apixaban and rivaroxaban.

POLICY

It is the policy of System Pharmacy & Therapeutics Committee to ensure appropriate usage and administration of Andexanet alfa. Refer to Attachments A-C for guidelines on how to achieve this goal.

SCOPE

This policy applies to all Northwell Health employees, as well as medical staff, volunteers, students, trainees, physician office staff, contractors, trustees and other persons performing work for or at Northwell Health; faculty and students of the Donald and Barbara Zucker School of

Medicine at Hofstra/Northwell or the Hofstra Northwell School of Nursing and Physician Assistant Studies conducting research on behalf of the Zucker School of Medicine on or at any Northwell Health facility.

DEFINITIONS

N/A

PROCEDURE/GUIDELINES

For patients with life-threatening bleeding: Refer to Attachment A titled *Andexanet alfa (Andexxa®) for Reversal of Oral Factor Xa Inhibitors due to Life-threatening Bleeding*

For patients requiring emergency neurosurgical procedures: Refer to Attachment B titled *Andexanet alfa (Andexxa®) for Reversal of Oral Factor Xa Inhibitors Requiring Emergency Neurosurgical Procedures*

SURVEILLANCE: The utilization of Andexanet will be reviewed and monitored by the CLOT Council for appropriateness of indications and adverse outcomes. In addition, the hospital's medical director, assistant executive director of finance and pharmacy director will receive email notifications of all Andexanet orders relevant to their sites.

CLINICAL REFERENCES

1. Andexanet alfa (Andexxa) [package insert]. Portola Pharmaceuticals, Inc. South San Francisco, CA. 2018.
2. Andexanet alfa. Lexi-Drugs. Lexicomp. Wolters Kluwer Health, Inc. Riverwoods, IL. Available at http://online.lexi.com/lco/action/doc/retrieve/docid/patch_f/6640361. Accessed April 24th, 2019.
3. Connolly SJ, Crowther M, Eikelboom JW, et al. Full Study Report of Andexanet Alfa for Bleeding Associated with Factor Xa Inhibitors. *N Engl J Med*. 2019;380(14):1326-1335.
4. Culbreth SE, Sylvester KW, Rimsans J, Connors JM. Coordinating emergent procedures after andexanet alfa. *Am J Hematol*. 2019;94(10):E278-E282.
5. Sherrod BA, Condie CK, Brock AA, et al. Emergent reversal of direct oral anticoagulants permitting neurosurgical intervention for nonhemorrhagic pathology. *World Neurosurg*. 2020;135:38-41.

REFERENCES to REGULATIONS and/or OTHER RELATED POLICIES

N/A

ATTACHMENTS

Attachment A: *Andexanet alfa (Andexxa®) for Reversal of Oral Factor Xa Inhibitors due to Life-threatening Bleeding*

Attachment B: *Andexanet alfa (Andexxa®) for Reversal of Oral Factor Xa Inhibitors Requiring Emergency Neurosurgical Procedures*

Attachment C**:** *Andexanet Administration Guide*

FORMS

N/A

**APPROVAL:**	
Northwell Health Policy Committee	❖6/28/2021
System PICG/Clinical Operations Committee	❖6/28/2021

Standardized Versioning History:
Approvals: * =Northwell Health Policy Committee; ** = PICG/Clinical Operations Committee; ✠ = Provisional; ❖ = Expedited
❖1/6/2020

Andexanet alfa (Andexxa) for Reversal of Oral Factor Xa Inhibitors due to Life-threatening Bleeding

INDICATIONS FOR USE:

1. Patients with apixaban or rivaroxaban exposure within 24 hours of ANY of the following life-threatening bleed:

   REQUIRE stroke neurologist/neurologist caring for stroke patients, neurointensivist, or neurosurgery approval for:
   - Acute intracranial hemorrhage
     i. If requiring emergency neurosurgical procedure, proceed to Attachment B: *Andexanet alfa (Andexxa®) for Reversal of Oral Factor Xa Inhibitors Requiring Emergency Neurosurgical Procedures*

   REQUIRE approval from the site medical director or designee:
   - Intraocular hemorrhage with vision compromise
   - Spinal or epidural hemorrhage
     i. If requiring emergency neurosurgical procedure, proceed to Attachment B: *Andexanet alfa (Andexxa®) for Reversal of Oral Factor Xa Inhibitors Requiring Emergency Neurosurgical Procedures*
   - Airway or pulmonary hemorrhage
   - Hemopericardium
   - Aortic rupture, dissection, or hemorrhage
   - Closed space hemorrhage- compartment syndrome risk

2. Andexanet alfa (Andexxa) is NOT INDICATED for bleeding from all other sites (including gastrointestinal bleeding and vaginal bleeding). For such patients, prothrombin complex concentrate (Kcentra®) is available. Please refer to Northwell CLOT Guidelines: Anticoagulation Reversal and Management of Bleeding Complications.

ANDEXANET HAS NOT BEEN STUDIED IN

1. Patients taking FXa inhibitors other than rivaroxaban or apixaban (i.e. edoxaban, betrixaban, enoxaparin)
2. Patients requiring reversal prior to urgent or emergent surgery
   - Patients with an acute life-threatening bleed meeting the indications for use may require surgical/procedural intervention as a co-management strategy. However, there is a concern for rebound bleeding/hypercoagulability, unpredictable pharmacokinetics and a lack of data in the surgical population. In contrast, PCCs have been studied in the surgical population.

3. Patients who have already received 4-factor prothrombin complex concentrate (i.e. Kcentra®, FEIBA®)

   - It is NOT recommended to co-administer andexanet (Andexxa) and Kcentra

DOSING

1. Dosing is based on the specific FXa inhibitor used, the FXa inhibitor dose, and the time interval since the last dose
2. Vitamin K (Phytonadione) is not indicated for reversal of FXa inhibitor-associated bleeds

Andexanet Dose Based on Apixaban or Rivaroxaban Use			
**Factor Xa Inhibitor**	**Last Dose of Factor Xa Inhibitor**	**Timing of FXa Inhibitor Last Dose Before Andexanet Initiation**	
		**Less than (<) 8 hours or Unknown**	**Greater tha or Equal to ≥ 8 hours**
Rivaroxaban	10 mG or 2.5 mG	Low dose	Low dose
Rivaroxaban	20 mG, 15 mG or Unknown	High dose	
Apixaban	5 mG or 2.5 mG	Low dose	
Apixaban	10 mG or Unknown	High dose	

Dose*	Initial IV Bolus	Follow-up IV Infusion
Low Dose	400 mG (~30 mG/min)	480 mG at rate of 4 mG/min for 120 min
High Dose	800 mG (~30 mG/min)	960 mG at rate of 8 mG/min for 120 min

*The safety and efficacy of an additional dose has not been established

PRECAUTIONS

1. Thrombotic events have been reported with the use of Andexanet
2. Incomplete reversal of anticoagulant activity may occur
3. Anti-FXa activity re-elevation: There is a rapid and substantial decrease in anti-FXa activity corresponding to the bolus dose, which is sustained during the continuous infusion. Anti-FXa activity returns to placebo levels ~2 hours after completion of bolus or continuous infusion; thereafter, anti-FXa activity decreases at a rate similar to the clearance of FXa inhibitors.
4. Andexanet has been reported to interfere with indirect anti-Xa inhibitors such as unfractionated heparin, which may result in ineffective anticoagulation.
5. Infusion related reactions occur in 18% of Andexanet-treated healthy volunteers vs 6% of placebo-treated subjects. See MONITORING for more information.

REINITIATION OF ANTITHROMBOTIC THERAPY

Reversing rivaroxaban and apixaban therapy exposes patients to the thrombotic risk of their underlying disease. Resume anticoagulant therapy as soon as medically appropriate.

PREPARATION, DISPENSING and STORAGE

RECONSTITUTION

A. Number of vials needed is dependent on low vs high dose regimen

- **Low dose**
  - BOLUS: 400 mG (two 200 mG vials) = 40 mL
  - Follow-up INFUSION: 480 mG (three 200 mG vials) = 48 mL
- **High dose**
  - BOLUS: 800 mG (four 200 mG vials) = 80 mL
  - Follow-up INFUSION: 960 mG (five 200 mG vials) = 96 mL

B. To reconstitute vials: (prepare all at once to reduce reconstitution time)

- Each 200 mG vial is reconstituted with 20 mL SWFI (10 mG/mL concentration)

1. Slowly inject SWFI onto the inside wall of the vial
2. Gently swirl vial until complete dissolution of powder. Do NOT shake.
3. Average dissolution time is 3 to 5 minutes per vial
4. Do NOT use vial if dissolution is incomplete – discard
5. Save all vials until drug is administered in the event that replacement request may be needed

C. To prepare IV bolus and Follow-up intermittent infusion:

1. Use a 60 mL syringe with a greater than or equal to (≥) 20 gauge needle to withdraw reconstituted solution from the vials
2. Transfer solution into an empty polyolefin or polyvinyl chloride IV bag with a volume of less than or equal to (≤) 250 mL
3. Prepare and dispense bolus and infusion doses in two separate IV bags
4. Save all vials until drug is administered in the event that replacement request may be needed

DISPENSING

1. The pharmacy will prepare and dispense in a ready-to-administer form in two separate IV bags (bolus and follow-up intermittent infusion)
2. The pharmacy will dispense with attachment C: Andexanet Administration Guide
3. The pharmacy will deliver andexanet or a representative will pick up andexanet from the pharmacy. It is a protein and cannot be sent through the pneumatic tube system.

STORAGE & STABILITY

1. Andexanet vials will be stored in the Pharmacy Department
2. Store intact vials at 2°C to 8°C (36°F to 46°F). Do not freeze.
3. Reconstituted vials are stable at room temperature for ≤ 8 hours, or may be stored for less than or equal to (≤24) hours at 2°C to 8°C
4. Reconstituted solution in IV bag is stable at room temperature for less than or equal to (≤) 8 hours, or may be stored for up to 16 hours at 2°C to 8°C.

RETURN TO MANUFACTURER

Andexanet unusable or expired vials, and unused IV bag(s) may be returned to the manufacturer for replacement request by following the Andexanet return procedure.

ADMINISTRATION and MONITORING

ADMINISTRATION

1. May be given in any patient care area for urgent/emergent use.
2. The bolus and infusion will be prepared together in 2 separate bags.
3. Do NOT discard unused bag(s) of Andexanet. Must return unused product(s) to the pharmacy department. Ensure patient's information is available along with reasoning for non-administration.
4. Administer Andexanet quickly and without delay.
5. Do not mix with other medicinal products. DO NOT infuse via same line with blood or other medicinal products.
6. Use 0.2 or 0.22 micron in-line polyethersulfone or equivalent low protein-binding filter for the BOLUS dose and follow-up INFUSION dose.
7. Program the Alaris pump by selecting Andexanet (if available) to match the medication order.
8. Administer the BOLUS dose first. Ensure to start the follow-up INFUSION dose **within 2 minutes** of completion of bolus dose.
9. Doses and rates of Andexanet administration in table below:

Andexanet Dosing Regimen	Initial IV Bolus	Follow-up IV Infusion
**Low Dose**	400 mG (40 mL) over 13 mins (180 mL/Hr)	480 mG (48 mL) at rate of 4 mG/min for 120 min (24 mL/Hr)
**High Dose**	800 mG (80 mL) over 27 mins (180 mL/Hr)	960 mG (96 mL) at rate of 8 mG/min for 120 min (48 mL/Hr)

Instructions for Administration

a. Inspect the bags visually for particulate matter and discoloration prior to administration, whenever solution and container permit.
b. Scan the barcode and check the label on the Andexanet IV bag
c. Flush the IV access line (may be pre-existing) with sterile 0.9% Sodium Chloride Injection, USP solution prior to infusion.
d. Spike the IV bag containing Andexanet with appropriate IV pump tubing
e. Prime tubing with solution in IV bag until no air remains.
f. Attach 0.2 or 0.22 micron in-line filter, piggy-back into main IV line, and attach to IV infusion pump.
g. Administer Andexanet at a standard infusion rate listed above and within medication order via IV infusion pump.
h. Stop infusion when IV bag is completely empty and unspike empty bag from tubing.
i. At the end of both bolus dose and infusion dose, flush the IV access line with at least 25 mL of 0.9% Sodium Chloride USP 50 mL bag at same rate. This will ensure that all content of IV bag is administered to patient.
j. When administration is completed, remove IVPB bag and tubing from IV access site and IV pump.

10. **Inter-Hospital Transfer**
    a. Administer BOLUS dose as described in the administration section.

    b. Prior to EMS transfer, start follow-up INFUSION dose to ensure medication is being administered during the transfer.

NURSING CARE

1. For patients in the Emergency Department (ED), ED Holding and Critical Care Units: Monitor as ordered by Physician, Nurse Practitioner and/or Physician's Assistant.
2. For admitted patients NOT already in ED, ED Holding and Critical Care Units: Initiate Rapid Response in order to provide appropriate monitoring.

MONITORING

1. Reactions were characterized by a range of symptoms
    a. Infusion reactions: fever, rigors, severe chills, hypertension, oxygen desaturation, agitation, confusion, etc.
    b. Flushing, feeling hot, cough, dysgeusia (distortion of the sense of taste), and dyspnea
2. Assess for signs and symptoms of adverse reactions:
    a. After starting BOLUS dose
    b. Immediately after the end of the BOLUS dose
    c. After starting the follow-up intermittent INFUSION
3. If patient develops severe infusion related reactions, stop the infusion and contact prescriber immediately
4. Observe for and report infusion-related reactions through local processes

Andexanet alfa (Andexxa®) for Reversal of Oral Factor Xa Inhibitors Requiring Emergency Neurosurgical Procedures

Indications:

- Emergency Procedure
  - Craniotomy
  - Spinal cord decompression
  - Burr hole
  - Extraventricular drain placement (EVD)*

*except in cases undergoing immediate angiography with the anticipation of using heparin during an endovascular procedure (e.g. stent placement) due to decreased patient responsiveness to heparinization

Procedure/Guideline:

1) Is the patient on apixaban or rivaroxaban (last dose taken less than (<) 60 hours ago)?
   a. **Yes**: Discuss case with neurointensivist attending, Director of Neurotrauma, or neurosurgery Chief of Service.
      i. Obtain and review PT/INR and LMWH-based anti-Xa level, when feasible
   b. **No**: If last dose apixaban or rivaroxaban greater than or equal to (≥) 60 hours ago (greater than on equal to 2.5 days), andexanet not indicated
2) Transport patient to appropriate setting (NSCU or OR) for procedure or operative intervention.
3) Begin initial andexanet bolus with the start of the procedure as per **Table 1 and Table 2** (below) based on the dose and timing of the last dose of apixaban/rivaroxaban.
   a. Bolus completion takes approximately 13 minutes and 27 minutes for the low and high dose, respectively
   b. Every attempt should be made to time administration so that procedural incision occurs upon completion or just prior to completion of bolus
4) Initiate andexanet follow-up infusion immediately after completion of initial andexanet bolus
   a. If surgical procedure time exceeds 4-hours after commencement of infusion, then further bleeding may be treated by administering Kcentra 50 units/kg approximately 2 hours after completion of andexanet infusion
5) Refer to ATTACHMENT A for additional information on preparation, dispensing, storage, administration, and monitoring

NOTES:

- There is *no direct contraindication* to andexanet for patients requiring emergent neurosurgical procedures, however this population has not been studied in clinical trials

- As approximately 10% of patients in clinical trials experienced a thrombotic event within 30 days of andexanet, all neurosurgical patients who have received andexanet should be closely monitored for arterial and venous thromboembolic events

Table 1

Andexanet Dose Based on Apixaban or Rivaroxaban Use			
**Factor Xa Inhibitor**	**Last Dose of Factor Xa Inhibitor**	**Timing of FXa Inhibitor Last Dose Before Andexanet Initiation**	
		**Less than (<) 8 hours or Unknown**	**Greater than or equal to (≥) 8 hours**
Rivaroxaban	10 mG or 2.5 mG	Low dose	Low dose
Rivaroxaban	20 mG, 15 mG or Unknown	High dose	
Apixaban	5 mG or 2.5 mG	Low dose	
Apixaban	10 mG or Unknown	High dose	

Table 2

Andexanet Dosing Regimen	Initial IV Bolus	Follow-up IV Infusion
**Low Dose**	400 mG (40 mL) over 13 mins (180 mL/Hr)	480 mG (48 mL) at rate of 4 mG/min for 120 min (24 mL/Hr)
**High Dose**	800 mG (80 mL) over 27 mins (180 mL/Hr)	960 mG (96 mL) at rate of 8 mG/min for 120 min (48 mL/Hr)

ANDEXANET ADMINISTRATION GUIDE

INDICATION

- FDA approved indication: urgent reversal of apixaban and rivaroxaban in adult patients with acute major bleeding
- For additional information regarding Andexanet use at Northwell Health, refer to system policy titled ***Andexanet alfa (Andexxa®) for Reversal of Oral Factor Xa Inhibitors due to Life-threatening Bleeding***

ADMINISTRATION

1. May be given in any patient care area for urgent/emergent use
2. The bolus and infusion will be prepared together in 2 separate bags
3. Do NOT discard unused bag(s) of Andexanet. Must return unused product(s) to the pharmacy department. Ensure patient's information is available along with reasoning for non-administration.
4. Administer Andexanet quickly and without delay
5. Do not mix with other medicinal products. DO NOT infuse via same line with blood or other medicinal products.
6. Use 0.2 or 0.22 micron in-line polyethersulfone or equivalent low protein-binding filter for the BOLUS dose and follow-up INFUSION dose
7. Program the Alaris pump by selecting Andexanet, if available, to match the medication order
8. Administer the BOLUS dose first. Ensure to start the follow-up INFUSION dose **within 2 minutes** of completion of bolus dose
9. Doses and rates of Andexanet administration in table below:

Andexanet Dosing Regimen	Initial IV Bolus	Follow-up IV Infusion
**Low Dose**	400 mG (40 mL) over 13 mins (180 mL/Hr)	480 mG (48 mL) at rate of 4 mG/min for 120 min (24 mL/Hr)
**High Dose**	800 mG (80 mL) over 27 mins (180 mL/Hr)	960 mG (96 mL) at rate of 8 mG/min for 120 min (48 mL/Hr)

10. **Instructions for Administration**
    a. Inspect the bags visually for particulate matter and discoloration prior to administration, whenever solution and container permit
    b. Scan the barcode and check the label on the Andexanet IV bag
    c. Flush the IV access line (may be pre-existing) with sterile 0.9% Sodium Chloride Injection, USP solution prior to infusion
    d. Spike the IV bag containing Andexanet with appropriate IV pump tubing
    e. Prime tubing with Andexanet solution until no air remains

f. Attach 0.2 or 0.22 micron in-line filter, piggy-back into main IV line, and attach to IV infusion pump
g. Administer Andexanet at a standard infusion rate listed above and within medication order via IV infusion pump
h. Stop infusion when IV bag is completely empty and unspike empty bag from tubing
i. At the end of both bolus dose and infusion dose, flush the IV access line with at least 25 mL of 0.9% Sodium Chloride USP 50 mL bag at same rate. This will ensure that all content of IV bag is administered to patient.
j. When administration is completed, remove IV bag and tubing from IV access site and IV pump

11. **Inter-Hospital Transfer**
    a. Administer BOLUS dose as described in the administration section
    b. Prior to EMS transfer, start follow-up INFUSION dose to ensure medication is being administered during the transfer

NURSING CARE

1. For patients in the Emergency Department (ED), ED Holding and Critical Care Units: Monitor as ordered by Physician, Nurse Practitioner and/or Physician's Assistant.
2. For admitted patients NOT already in ED, ED Holding and Critical Care Units: Initiate Rapid Response in order to provide appropriate monitoring.

MONITORING PARAMETERS

1. Reactions were characterized by a range of symptoms
    a. <u>Infusion reactions:</u> fever, rigors, severe chills, hypertension, oxygen desaturation, agitation, confusion, etc.
    b. Flushing, feeling hot, cough, dysgeusia (distortion of the sense of taste), and dyspnea
2. Assess for signs and symptoms of adverse reactions:
    a. After starting BOLUS dose
    b. Immediately after the end of the BOLUS dose
    c. After starting the follow-up intermittent INFUSION
3. If patient develops severe infusion related reactions stop the infusion and contact prescriber immediately
4. Observe for and report infusion-related reactions through facility processes
